# Cultural adaptation and feasibility analysis of What Were We Thinking (WWWT): A psychoeducational intervention to prevent emotional disorders in fisrt-time mothers

**DOI:** 10.1192/j.eurpsy.2026.11848

**Published:** 2026-07-31

**Authors:** E. Gelabert, N. Fernández-Jarabo, A. Torres-Gimenez, S. Andrés-Perpiñá, C. Martínez-Bueno, L. Garcia-Esteve, S. Subirà, A. Roca-Lecumberri

**Affiliations:** 1 Departament of Clinical and Health Psychology, Universitat Autònoma de Barcelona, Bellaterra (Barcelona); 2 Perinatal Mental Health Unit CLINIC-BCN, Hospital Clínic; 3 Direcció Assistencial d’Atenció Primària. Servei d’Atenció a la Salut Sexual i Reproductiva (ASSIR), Institut Català de la Salut, Barcelona, Spain

## Abstract

**Introduction:**

Depression and anxiety are prevalent among first-time mothers. Evidence suggests that culturally adapted psychoeducational interventions can effectively prevent perinatal emotional disorders when integrated into local health care systems.

**Objectives:**

This study aimed to culturally adapt *What Were We Thinking* (WWWT, Fisher et al., BMC Public Health 2010;10:1), an Australian gender-informed psychoeducational program for couples with newborns, to the Catalan primary health care context, and to assess the feasibility of the adapted intervention.

**Methods:**

Cultural adaptation followed a structured model, including literature review, stakeholder consultations, translation, expert review, and pilot testing guided by the Ecological Validity Model (Bernal et al., Prof Psychol 2009;40:361–8). A qualitative pilot study (n=13) explored acceptability, usefulness, and comprehensibility. A quantitative feasibility analysis was then conducted within a randomized controlled trial involving 60 participants who received the intervention, assessing recruitment, adherence, retention, and participants’ perceptions of comprehensibility, acceptability, and relevance.

**Results:**

Adaptations improved cultural relevance, language clarity, and contextual fit. The pilot study indicated that the program was understandable, meaningful, and relevant. Preliminary quantitative analysis of the RCT sample suggested satisfactory recruitment, high session attendance, low attrition, and positive perceptions of comprehensibility, acceptability, and relevance, supporting feasibility in the Catalan primary care context.

**Conclusions:**

The Catalan and Spanish versions of WWWT represent culturally sensitive adaptations of the original program. Qualitative and quantitative findings provide preliminary evidence of feasibility and acceptability, supporting the rationale for a larger-scale trial to evaluate effectiveness.

This study was supported by funding from the Fundació La Marató.

**Disclosure of Interest:**

E. Gelabert Grant / Research support from: Fundació la Marató: 193/C/2022, N. Fernández-Jarabo: None Declared, A. Torres-Gimenez: None Declared, S. Andrés-Perpiñá: None Declared, C. Martínez-Bueno: None Declared, L. Garcia-Esteve: None Declared, S. Subirà: None Declared, A. Roca-Lecumberri Grant / Research support from: Fundació la Marató: 193/C/2022

